# Spinal manipulation under anesthesia: a narrative review of the literature and commentary

**DOI:** 10.1186/2045-709X-21-14

**Published:** 2013-05-14

**Authors:** Dennis DiGiorgi

**Affiliations:** 1 DC, CHCQM, CCIC, Consultant Practice- Whitestone, New York, USA

**Keywords:** Manipulation under anesthesia, Spine, Efficacy, Medical evidence, Quality, Ethics

## Abstract

As exhibited throughout the medical literature over many decades, there is a lack of uniformity in the manner in which spine pain patients have historically qualified for and received manipulation under anesthesia (MUA). Also, for different professions that treat the same types of spinal conditions via the same means, fundamental MUA decision points vary within the published protocols of different professional associations. The more recent chiropractic literature communicates that the evidence to support the efficacy of MUA of the spine remains largely anecdotal. In addition, it has been reported that the types of spinal conditions most suitable for MUA are without clear-cut consensus, with various indications for MUA of the low back resting wholly upon the opinions and experiences of MUA practitioners. This article will provide a narrative review of the MUA literature, followed by a commentary about the current lack of high quality research evidence, the anecdotal and consensus basis of existing clinical protocols, as well as related professional, ethical and legal concerns for the chiropractic practitioner. The limitations of the current medical literature related to MUA via conscious/deep sedation need to be recognized and used as a guide to clinical experience when giving consideration to this procedure. More research, in the form of controlled clinical trials, must be undertaken if this procedure is to remain a potential treatment option for chronic spine pain patients in the chiropractic clinical practice.

## Background

MUA has been reported in the medical literature since the 1930’s [1]. Inspection of the literature reveals that medicine assisted manipulation (MAM) [2], across its varied forms- manipulation under general anesthesia or conscious/deep sedation (MUA), manipulation under joint anesthesia (MUJA) or manipulation under epidural anesthesia/epidural steroid injection (MUEA/MUESI)- has been used to treat a host of musculoskeletal conditions [1,3-30]. Table 1 summarizes many of the clinical diagnoses traditionally reported and treated by MAM.

**Table 1:** Musculoskeletal conditions treated with medicine assisted manipulation (MAM)

		
**Cervical spine**		**Thoracic spine**
Cervical disk herniation/syndrome [4,8]		Intractable intercostal neuritis [10]
Cervical pain [3,5-7]		Thoracic pain [5,7]
Cervical radiculopathy [4,9]		
Cervicogenic headache [3,4]		**Other**
Traumatic torticollis [10]		Acute muscle spasm with subluxation [27]
**Lumbar spine/pelvis**		Acute osteopathic lesion pathology [17]
Acute low back pain [7]	Lumbar intervertebral disc syndrome [17,25,26]	Acute psoasitis [17]
Arthritic changes of the low back [17]	Lumbarization/sacralization [10]	Chronic muscle contracture [6,27]
Chronic low back pain [5,11-15]	Lumbar nerve root compression syndrome [23,24]	Chronic myositis [27]
Chronic lumbosacral strain [29]	Lumbar post-laminectomy syndrome [22]	Extremity dysesthesias related to cervical or lumbar pain [5]
Chronic sacroiliac strain [1,27]	Lumbar radiculopathy [9]	Fibrosis/(myo)fibrositis [10,17,27,28]
Degenerative lumbar scoliosis [19]	Lumbosacral disc protrusion [11]	Nerve entrapment [27]
Disturbed lumbar disc integrity [10]	Postural defects of the low back [20]	Old compression fractures [10]
Failed back surgery syndrome [12,21]	Recalcitrant synovial joint mediated low back pain [18]	Osteoporosis [10]
Joint stiffness of the low back [17]	Rigidity of the low back [20]	
Low back pain with leg weakness and/or pain [11,12,16]	Spondylolisthesis [17,30]	
Lumbar disc derangement [30]	Spondylosis [30]	

Historically, there has remained a strong theoretical basis for the application of MUA to the axial spine and associated soft tissues. It has been proposed that by disrupting or stretching adhesions [4,12,20,25,31,32] a restoration of articular mechanics can be realized [4,10,12,32,33]. Nevertheless, the most recent review paper on medicine assisted manipulation for chronic low back pain communicates that the theories that MUA more effectively treats adhesions and that adhesion reduction increases flexibility are without the support of experimental research [2]. Of equal inference is the notion that these theories cannot be contested absent such research [2]. Thus, there is a void of medical evidence to either confirm or deny the validity of the principal clinical basis for utilizing spinal MUA.

The addition of anesthesia to the manipulative procedure serves to eliminate pain inhibiting reflexes and to allow for relaxation of muscles so that treatment can be delivered more effectively [10,34]. Essentially, MUA of the spine is intended for use with two general categories of pain conditions [32,35], and when manipulation is the therapeutic procedure of choice [35]:

### The acute condition (i.e., acute onset of a recurrent condition)

MUA may be pursued when a patient’s pain is so intense and debilitating that medication management and/or the application of standard chiropractic treatment is precluded [35,36]. Immediate relief is desired in an attempt to have the individual return to pre-injury status as soon as possible [35]. In the chiropractic literature it has been reported that MUA is not usually applied in cases of acute trauma [35], but if so, only a single procedure dose would typically be required to return the patient to office-based care [32]. Aside from the single procedure dose approach it has also been reported that the application of intermittent (non-consecutive) MUA procedure doses may be justified in the treatment of acute musculoskeletal conditions [37].

### The chronic condition

For the chronic condition MUA is indicated when a patient’s pain has proven to be of limited responsiveness in part to trials of traditional office-based manipulative procedures (over a period of weeks [33,35,37]), and when the condition has a measurable detrimental impact upon functionality [5]. Treatment is directed at eliminating the fibrotic adhesions presumed responsible for altering one’s ability to engage in routine activities versus pre-injury or pre-condition levels. In qualifying the extent to which physical incapacitation may warrant the use of MUA it has been depicted that condition intensity can render “impossible” patient engagement in therapeutic exercise [38]. The procedure may be most appropriate once other modes of conservative care have been exhausted and the final patient decision scenario of surgery versus MUA is reached [38].

In 1992, Greenman [6] reported that the need for MUA is “not common”. Elsewhere, it has been suggested that only a small minority of patients with musculoskeletal disorders/mechanical dysfunctions will require the like, perhaps spanning from 3% to 10% [5,7]). However, these figures on patient candidacy have yet to be validated by way of controlled investigation [2], thereby suggesting lack of substantiating evidence for them.

The MUA procedure has evolved considerably since initially reported in the early osteopathic literature. This has been acknowledged by chiropractic investigators [2,34]. Older papers describe or imply the rendition of mostly a single MUA procedure dose by osteopathic/medical physicians with an involved patient hospital stay [7,17,25,27,28]. Nowadays, MUA of the spine is usually administered in serial fashion [5,8,31], on an outpatient basis, with the principal provider type being chiropractors [39]. Paralyzing anesthetic drugs are no longer in use, while various types or combinations of hospital-based co-interventions are not part of the contemporary treatment regimen (i.e., shortwave diathermy [20], intramuscular medication [20], intramuscular vitamin E [20], muscle relaxants [17], vitamin B6 [17], various forms of traction [7,17,20,26-28,40] and fitted back brace [26]). The contributing role of any or all of the early methods in the study outcomes previously reported is not known.

From an historical perspective, the eventual participation of chiropractors in spinal MUA occurred many years after orthopedic manipulation had fallen by the wayside and only after the larger osteopathic community hadn’t taken acceptance to the MUA procedure [34]. One might deduce that an absence of perceived treatment efficacy for MUA was the principal causative factor for its generalized lack of popularity amongst allopathic physicians. As such, the contemporary chiropractic clinician should not rely upon decades old clinical papers, which cite a distinctly different MUA treatment regimen from that in use today, as an unconditional or rote support basis for MUA of the spine via conscious/deep sedation.

Proponents of the MUA procedure once categorized it as a last resort treatment option for those facing surgical intervention [38]. Nonetheless, under the domain of chiropractors MUA has arguably become a mode of care commonly administered under far less pressing clinical circumstances and with growing frequency. There is a general lack of published outcomes data in the peer reviewed medical literature to explain or support this element of the evolutionary process. Moreover, the manner in which the post-MUA therapy and rehabilitation component of care may contribute to the patient improvement claims frequently made by chiropractors is not known.

### Review of the literature

Reviewing the medical literature on spinal manipulation under anesthesia presents a significant challenge on account of lack of a comparative nature of the procedure, and related components, over the course of many decades. Most published clinical studies on medicine assisted manipulation reflect largely positive outcomes. As such, some might consider MAM a universal treatment strategy for appropriately selected patients with spine-based musculoskeletal pain or disability. Nonetheless, by applying the levels of evidence schema introduced nearly a decade ago by Wright et al. [41] as a method to rate the more commonly cited or relied upon published clinical studies on MAM, the quality of research evidence can be gauged by way of a contemporary standard (Table 2). It is through this process that the lack of high quality supportive scientific evidence for spinal MUA is revealed.

**Table 2:** Synopsis of the routinely cited or reviewed published research papers on MAM of the spine

MUA or MAM*				
**Author(s)**	**Publication year**	**No. of procedure doses**	**Study design**	**Level of research evidence**^**†**^
Kohlbeck FJ, et al. [13]	2005	1 to 3 (over consecutive weeks)	Cohort study (prospective)	II
Palmieri and Smoyak [15]	2002	1 to 4 (over the same number of days)	Cohort study (prospective)	II
Siehl D, et al. [23]	1971	1	Cohort study/RCT ^**#**^	II
Morningstar and Strauchman [21]	2012	3 (over consecutive days)	Case series	IV
Morningstar and Strauchman [19]	2010	3 (over consecutive days)	Case report ^**+**^	IV
Cremata E, et al. [5]	2005	3 (over consecutive days)	Case series	IV
Herzog J [4]	1999	3 (over consecutive days)	Case report	IV
West DT, et al. [31]	1999	3 (sequentially)	Case series	IV
West DT, et al. [42]	1998	3 (sequentially)	Case series	IV
Davis CG [3]	1996	At least 1 and up to 3 (consecutively or intermittently)	Case series	IV
Alexander GK [22]	1993	5 (serial)	Case report	IV
Davis CG, et al. [12]	1993	3 (over consecutive days)	Case reports	IV
Hughes BL [8]	1993	3 (daily basis)	Case report	IV
Greenman PE [6]	1992	1	Case report	IV
Chrisman OD, et al. [25]	1964	1	Case series	IV
Siehl, D [28]	1963	1 (91%), 2 or more (9%)	Case series	IV
Bremner, RA [29]	1958	1	Case series	IV
Mensor MC [26]	1955	1 (83%), 2 (17%)	Case series	IV
Soden CH [10]	1949	1	Case reports	IV
**MUEA/MUESI or MUJA**^**ℓ**^				
Dougherty P, et al. [9]	2004	1 (67.5%), 2 (25%), 3 (6.25%), 4 (1.25%)	Case series	IV
Nelson L, et al. [14]	1997	1	Case series	IV
Aspegren DD, et al. [16]	1997	1	Case reports	IV
Dreyfuss P, et al. [18]	1995	1 or 2	Case reports	IV
Ben-David and Raboy [11]	1994	1	Case reports	IV
Warr AC, et al. [30]	1972	1	Case series	IV
Haldeman and Soto-Hall [1]	1938	1	Case series	IV

* With procedural application to one or more spinal regions via general anesthesia or conscious sedation.

^†^ When applying the levels of evidence rating system for categorizing study quality, as put forth by Wright, et al. [41] and adopted by the *Journal of Bone & Joint Surgery*[41], *Spine, Clinical Orthopaedics and Related Research*, the North American Spine Society, the American Academy of Orthopaedic Surgeons, and the Pediatric Orthopaedic Society of North America [43].

^+^ The case report study design has not been rated by Wright, et al. [41]. In terms of qualitative value it is likely most analogous to expert opinion (Level V evidence). However, in eliminating the appearance of bias toward underestimating its significance it has been coupled here with the case series study design and designated as Level IV evidence.

^#^ Within the medical literature this study has been classified differently, as a Cohort study [13,34] and as an RCT [2]. As the final paper from Siehl, et al. [23] does not specifically cite the element of patient randomization, the Cohort study design classification appears to be correct. Nevertheless, the results reported [23] pertain to 47 of 147 patients (less than 80% follow-up). Therefore, even as an RCT, this study would qualify as Level II evidence under the rating system put forth by Wright, et al. [41].

^ℓ^ With procedural application to specific spinal regions via MUEA/MUESI or MUJA (MAM agents applied locally).

Abbreviation key: *MAM*- medicine assisted manipulation, *MUA*- manipulation under anesthesia, *MUEA*- manipulation under epidural anesthesia, *MUESI*- manipulation under epidural steroid injection, *MUJA*- manipulation under joint anesthesia.

Many of the MAM studies within the medical literature are of the case report or case series variety. Namely, each of numerous published reports spanning from 1949 to 2012 [3-6,8,10-12,16,18,19,21,22] accounts for only a select few patients undergoing MUA or MUJA/MUEA (ranging from 1 to 5 subjects). However, case reports or small case series are of limited value in that they are typically comprised of only successful cases, and are descriptive in nature as opposed to analytic/experimental [44,45]. The MUA case series by Morningstar and Strauchman cites inherent bias with a retrospective patient selection process [21]. Consequently, the case report/series study design lies relatively low in the hierarchy of medical evidence and specific cause and effect relationships cannot be determined [46].

The MUJA/MUEA treatment related case reports or case series offered by Aspegren, et al. [16], Ben-David and Raboy [11], Dougherty, et al. [9], Dreyfuss, et al. [18], Haldeman and Soto-Hall [1], Nelson, et al. [14] and Warr, et al. [30] all cite favorable results. It has been suggested or hypothesized that the efficacy of the MUJA procedure, or proposed manipulation following periarticular anesthetics, may be related to facilitation of the manipulative maneuver [47,48]. Specific to MUEA, it has been postulated that observed treatment efficacy for radiculopathic conditions of the cervical or lumbar regions is related to the combined effect of addressing both the inflammatory and mechanical components of pain [9].

In the management of chronic lumbosacral strain, the results of the studies conducted by Bremner [29] and Bremner and Simpson [49] were compared in determining patient response to two different treatment methods [49]. In the earlier study of 250 patients, manipulation of the lumbar spine under general anesthesia was performed, followed by physiotherapy for two weeks [29]. In the latter study involving 150 patients treated via physiotherapy three times per week for four weeks, treatment was comprised of deep massage to the lumbosacral spine, manipulation, strengthening exercises and, in some cases, short-wave diathermy [49]. The percentages of patients showing any improvement were 86.8% and 87.4%, respectively. Both treatment methods, either with or without MUA, were deemed to offer an equally beneficial immediate result.

Significantly positive outcomes for pain, patient work status and medication use were reported in the large MUA retrospective case series conducted by West, et al. [31]. However, those results are of uncertain value due to confounding factors with the study design. Namely, patient selection was not limited by diagnosis while patients were generically grouped by cervical or lumbar conditions despite the number of symptomatic anatomic regions. Furthermore, MUA was rendered on a multi-regional basis for all patients rather than being directed at the region of primary diagnosis. Also, comparative post-MUA functional capacity outcomes data were generally collected six weeks after MUA, apparently only after the inception of an intensive post-MUA rehabilitation program. Last, and perhaps of greatest significance, this same study had been previously published, alternatively citing that 20 of the 177 patients in the treatment group were in receipt of “anesthetic/corticosteroid epidural injection” at the outset of MUA treatment for sequestered disc herniation [42]. The more recent West paper [31] offers no mention of this and does not address the potential therapeutic impact of the injection on the group of subjects that had received it relative to those who underwent MUA (conscious sedation) alone.

The prospective cohort studies undertaken by Kohlbeck, et al. [13] and Palmieri and Smoyak [15] refer to 42 and 38 subjects, respectively, in receipt of single or serial MAM/MUA for chronic low back pain versus a control group. Between these two studies there are variations in technique application, the span of time between any serially administered procedure doses (consecutive days versus consecutive weeks), and the intravenous agents utilized. Although both clinical papers chronicle results that are encouraging (e.g., more improvement for the MAM/MUA treatment group in the patient-perceived outcome categories of pain and disability), neither study was conducted by way of a randomized trial. Both sets of authors acknowledge this fact and conclude that large-scale clinical studies (i.e., multi-site, randomized controlled trials) appear warranted in this area [13,15]. It should be noted that in the absence of randomization, it is significantly less likely that treatment and control groups will be balanced with regard to both the known and the unknown factors affecting outcome [46]. Therefore, while the results of each of these observational studies are both favorable and encouraging they are simply not conclusive enough to generalize that MAM or MUA via conscious sedation can be considered efficacious across the spectrum of chronic spine pain populations (low back or otherwise).

There is a general paucity of high quality clinical papers in the area of MUA management of intervertebral disc related conditions with a suspected neurological component of radiating pain into an extremity. In the presence of *EMG confirmed* lumbar nerve root compression, the study by Siehl, et al. [23] does not favor the use of MUA under that particular clinical circumstance. The authors of that paper opined that the trend of outcome deemed the procedure ineffective over the long term in the presence of positive EMG findings, with surgery likely required at some point. For lumbar disc herniation without EMG evidence of nerve root compression it was opined that MUA would probably offer lasting benefit [23]. Within the medical literature, this study has been alternately referred to as a Cohort study [13,34] and a randomized controlled trial [2]. Regardless of classification (both qualifying as Level II evidence), the findings of Siehl, et al. [23] were recently summarized in a literature synthesis put forth by the Scientific Commission of the Council on Chiropractic Guidelines and Practice Parameters [50]. Therefore, in the context of that seminal paper [23] it cannot be summarily assumed that absent electrodiagnostic testing, patient symptomatology of chronic lower back pain with a referred/radiating component into a lower extremity is necessarily indicative of a condition that may warrant or support consideration for MUA.

### Commentary about the literature

MUA has been classified as both “surgical” [10,51] and “nonsurgical” [2]. Regardless of classification, recent multidisciplinary expert panel reviews of the interventions for neck and low back pain conditions do not include an analysis of any form of medicine assisted manipulation [52-55]. One might argue that the overall lack of high quality studies in this area, for specific clinical diagnoses, renders MUA of the spine controversial despite its seeming widespread use and strong theoretical basis. In contrast, the utilization of MUA to treat certain extremity conditions (i.e., frozen articulations of the shoulder or knee) has likely earned a greater degree of acceptance amongst practitioners and third party payers alike due to a gradually mounting body of supportive medical evidence [56-61]. So, despite the presence of MAM in the medical literature for many decades, questions remain as to whether MUA via conscious/deep sedation can be considered a clinically authenticated treatment option for acute or chronic neck and low back pain conditions across varying etiologies.

More than a decade ago an opinion paper cited that more than 20,000 patients in the US and the UK had received MUA since the late 1930’s [32]. Since the publishing of that paper, certainly the number of chiropractors in the United States attaining MUA certification has grown. Considering this, as well as increasing popularity and a greater degree of MUA utilization within the chiropractic profession over that period, the relative paucity of published studies in the peer reviewed medical literature represents a glaring void. To date, as part of the natural progression of clinical research [62], the MUA protocols routinely used by chiropractors have not been subjected to a single large-scale randomized controlled trial for any spinal condition or diagnosis so as to reveal the evidence of efficacy or in serving to support serial MUA over a single procedure dose.

Advocates of spinal MUA may find themselves in a compromised position when they ignore the void of scientific evidence for this procedure. For the treatment of spine-based musculoskeletal pain/dysfunction most major third party payers in the United States have designated MUA “experimental/investigational”. The sole basis for this unfavorable designation is the current lack of high quality evidence for MUA. However, these same payers take a favorable position with the allopathic version of MUA of the spine, when it involves the reduction of vertebral or pelvic fracture/dislocation [63-65]. The role of MUA in evaluating pelvic fracture stability following trauma has most recently been investigated [66].

The best evidence for MAM or MUA of the spine relates to the management of chronic low back pain (Level II evidence), as put forth in the controlled prospective cohort studies undertaken by Kohlbeck, et al. [13] and Palmieri and Smoyak [15]. However, these authors acknowledge the need for additional large scale studies in attaining more definitive data on treatment efficacy [13,15]. Consequently, the results of these studies should not be extrapolated as evidence of efficacy for MUA in treating different spine pain populations or when different agents/techniques from those outlined are implemented in similar spine pain populations. In terms of the vague nature of the manifestation diagnosis of pain (i.e., chronic low back pain), perhaps additional investigation would be beneficial in identifying specific clinical diagnoses of the low back that may be amenable to MUA. With regard to the treatment of EMG confirmed lumbar intervertebral disc related nerve root compromise, the only study undertaken to date [23] resulted in an outcome trend suggesting that MUA was ineffective over the long term (Level II evidence). Those same authors also opined that lasting improvement will probably be experienced in those with negative EMG-related low back pain with radiation to one or both legs.

There are no randomized controlled trials or published cohort studies on MUA management of specific diagnoses of the cervical or thoracic regions. As such, the efficacy of such treatment has yet to be adequately explored. As per the work of Krumhansl and Nowacek [38], despite a high percentage of favorable results attained for the 171 subjects treated by way of MUA for conditions of the lumbar and/or cervical regions, *not a single patient* received an extension of that care to the conjoining thoracic spine. Also, it was reported that relatively few (11%) of those same patients were in receipt of a second procedure dose. None required a third. In the large case series undertaken by Siehl, manipulation of the dorsal (thoracic) spine under general anesthesia was rendered “occasionally”, while 9% of patients required more than one procedure dose [28]. Thus, in order to determine the efficacy of MUA for primary conditions of the cervical and thoracic regions, and in clarifying the dosing thresholds necessary for best patient outcomes, diagnosis specific comparative studies are needed.

There is no published medical evidence to support the common approach of universal MUA treatment of the entire axial spine in the management of an isolated regional condition (i.e., recalcitrant lumbar pain, with disabling range-of-motion loss). This remains true even in the presence of secondary and relatively innocuous complaints/physical findings of vertebral joint pain/dysfunction of other spinal regions. In fact, as reported by Krumhansl and Nowacek, following a single MUA procedure to the lumbar region, corrective mobilization of the upper thoracic and cervical regions is usually attained with a rigorous three day manual therapy regimen [38]. Also, relative to an initial MUA procedure dose to the lumbar region, subsequent application of MUA to treat cervical spine injuries is required infrequently (with about 5% of cases). Reportedly, this holds true even for injuries associated with rear-end vehicular collisions (with 20% of those cases selected for MUA) [38].

The clinical value of the distinct application of MUA to the shoulder and/or hip articulations, as a natural extension of MUA treatment of approximating vertebral/pelvic joints, has yet to be determined through scientific investigation. It is recognized that some of the commonly applied spine-related MUA maneuvers/techniques rely on the upper or lower extremity as a long lever. This serves to stretch the musculature from origin to insertion as it traverses both the targeted vertebral/pelvic motion units under care and the conjoining extremity. However, technique application does not signify that any incidental or intentionally induced joint cavitation from the glenohumeral or femoroacetabular articulations is an integral component of care such that it provides additional therapeutic benefit to the patient’s treating spinal condition (whether or not there is an associated component of pain referral/radiation to the extremities). In fact, published MUA studies on the shoulder and hip joints are concerned solely with primary conditions of these articulations, such as adhesive capsulitis [57-59,67]. Consequently, any supportive medical evidence for the utilization of MUA to treat frozen shoulder or hip articulations does not serve as a clinical basis for the routine application of MUA to these extremity joints when rendered as an adjunctive form of care during the MUA management of a spine pain condition. This type of treatment approach has been criticized in the chiropractic literature [68].

There is a growing body of evidence on the use of MUA to treat frozen shoulder (adhesive capsulitis) [57-59] and post-operative fibroadhesions of the knee [60,61], when rendered as a single dose orthopedic procedure. However, a recent health technology assessment found limitations in the studies published on MUA management of frozen shoulder [69], with the only study deemed adequate revealing no evidence of better outcome with MUA over home exercise. For similar conditions of the hip joint (the femoroacetabular joint [67]), there is a general paucity of clinical papers in the peer reviewed medical literature.

### Clinical considerations

#### Clinical issues of patient selection

Although manipulation of the spine under anesthesia is currently in general use by chiropractic professionals, it is an advanced form of treatment [35] not intended as a first-line therapy or routine service. For each of the varied forms of MAM, treatment is reserved for individuals who have already pursued traditional modes of care [3-5,7,9,11,12,14-16,18,25],[31,33,36,38,47] (including, in part, spinal manipulation), but for whom the condition is recalcitrant [47]. Significant pain and dysfunction typically preclude a return to normal activities [5], whether personal, occupational or recreational. Acutely symptomatic conditions can be managed by MUA when immediate relief is desired but traditional modes of care including spinal manipulation are not tolerated [35] (i.e., with an acute idiopathic torticollis [36]). However, MUA is more commonly directed at the chronic and recalcitrant variety of musculoskeletal condition [32,38] which has not resolved as expected with conservative care or in accordance with the natural history of healing.

Descriptions of locked or immovable spinal joints have been offered as a primary patient qualifier for MUA [38,70,71]. This would suggest the presence of “a state of fixation” [71] by which the facet joint articulations of one or more vertebral motion units remain reflexogenically/biomechanically frozen or are bordering on pathological fusion. It is posited here that this level of vertebral joint “dysfunction” is seldom encountered in chiropractic practice.

It is well established that asymptomatic and/or atraumatic individuals can display positive findings upon magnetic resonance imaging of the cervical and lumbar regions [72-76], many of which are known phenomena of aging [77-79]. There is evidence that the anatomically mapped referral zones for neck and low back pain of sclerotomal and myotomal origin [80-85] can resemble or mimic patterns of radiating pain of dermatomal origin [86-90]. Both of these factors can confound the clinical picture when caring for patients with trauma induced spine pain conditions which include a referral/radiation component into an extremity. It’s known that absent inflammation, spinal nerve root compression on its own does not cause pain, although physical signs of motor, deep tendon reflex and/or sensory deficits can occur [91,92]. Therefore, in the context of the findings of Siehl, et al. [23], each of these factors must be taken into consideration when patients exhibiting the aforesaid symptom complex are being evaluated for MUA.

#### The joint cavitation phenomenon

What makes chiropractic care unique in the realm of existing conservative management options for spine pain is the skilled manipulation component of that care. When spinal joints are manually manipulated they are moved passively to their physiological limit before receiving a dynamic thrust which separates the articular surfaces [93], resulting in joint cavitation (an audible crack) [93,94]. Joint cavitation is the consequence of an immediate reduction of intra-articular pressure and the liberation of gases from the synovial fluid, and results in a transitory period of joint surface separation due to the presence of a newly formed gas bubble [93,94]. The gapping of synovial joint surfaces, or the temporary induction of joint buoyancy, likely plays a role in the relief of joint pain and/or stiffness.

It was previously reported that a potential association between the therapeutic benefits attained with spinal manipulation and the joint cavitation phenomenon had yet to be fully investigated [95]. More recently, it has been revealed that a reduction in erector spinae muscle spindle stretch reflex activity occurs *only* when spinal manipulation is accompanied by an audible release [96]. Albeit preliminary, this might suggest a biological mechanism to the pain reducing effects of spinal manipulation. Joint cavitation may serve to interrupt muscle spindle stretch reflex excitability, part of the pain-spasm-pain cycle [96]. The analgesic/hypoalgesic effects of spinal manipulation have been discussed elsewhere [93,97-101], as have the mechanical/physiological benefits of increased joint range of motion [91,93,100] and a reduction of articular adhesions [93].

### Clinical issues of manipulation technique

#### Thrust versus non-thrust techniques

Under the domain of chiropractic care lays numerous named spinal adjusting techniques [102-105], many of which are implemented with the intent of maneuvering synovial joints to the extent that cavitation is achieved. In theory, the audible release attained via different manipulation techniques could vary in terms of the side or vertebral level affected, with potential for better health outcomes upon modification of technique [106]. The Diversified technique is that which is most commonly utilized in chiropractic practice [107,108] and rendered with the clinical intent of eliciting joint cavitation. It is the only acceptable technique to utilize when delivering manipulations during the MUA procedure [35,109].

Two commonly utilized and well accepted chiropractic techniques that are applied without an explicit intent to elicit joint cavitation, on account of means of delivery, are the Activator Method and Cox Flexion Distraction. The former technique is administered by way of a handheld spring-loaded adjusting instrument that renders a low force impulse into spinal joints [110]. The latter technique is administered by way of a treatment table with break-away sections that allow multi-planar distractive forces to be applied principally to intervertebral discs [111,112]. Although mechanically assisted manipulation with an impulse device such as the Activator adjusting instrument is categorized as a high velocity, low amplitude procedure [50], flexion distraction methods are considered within the realm of mobilization [50]. Hence, patients who have not received chiropractic treatment via manual manipulation techniques aimed at inducing joint cavitation have not undergone a trial of care akin to that which is utilized during the MUA procedure. Accordingly, it is with a patient’s best interests in mind that adequate trials of in-office chiropractic manipulations should be comprised of one or another type of joint cavitation technique, assuming patient toleration, before the individual may be considered for potential placement into an MUA program.

#### Full spine versus regional manipulation

Many chiropractors adhere to a patient care ideology of treating the entire spine in achieving a state of structural and functional balance. However, for patients being managed by way of MUA, this philosophical precept is not supported by current medical evidence. As previously proffered by Krumhansl and Nowacek, corrective mobilization of the upper thoracic and cervical regions is usually attained with a rigorous three day manual therapy regimen following a single MUA procedure to the lumbar region [38]. Subsequent application of MUA to the cervical spine was reported to be infrequently required, even in cases of rear-end vehicular collisions [38]. As MUA is intended to be reserved for those exhibiting significant pain and dysfunction of a particular body region (which precludes normal activities [5]), the practice of full-spine application should not be routine but rather determined on a case-by-case basis with supportive clinical logic.

Treatment of a targeted spinal region via MUA necessitates the stretching of conjoining spinal regions incidental to the origin and insertion of the involved musculature. In and of itself, this does not constitute as MUA treatment of the secondary spinal region/s. Although conscious manipulation to a body region that conjoins another with pain or dysfunction can provide clinical benefit to the affected site [113-117], the evidence for this practice is limited and inconsistent [118]. That evidence should not be extrapolated to support the provision of multi-regional MUA care when treating a patient primarily for an isolated spinal condition. To the contrary, as reported by Krumhansl and Nowacek [38], evidence exists for the efficacy of short-term post-MUA office-based care in addressing secondary issues of spinal regions not treated via MUA.

#### MUA dosage

Divergent sets of protocols/indications for MUA exist [119,120] in part, with regard to the requisite conservative treatment timeframes associated with patient selection as well as procedure dose application. This raises questions as to what constitutes as the professional standard of care for MUA intervention and dosage. In the absence of a single and uniform process by which patients may qualify for and receive MUA it is easily inferred that the most fundamental decision points relied upon are lacking high quality supportive evidence. Instead, they rest upon consensus processes of different professional associations. Within the more recent chiropractic literature it has been said that the evidence to support the efficacy of MUA of the spine remains “largely anecdotal” [34], that various indications for MUA of the low back rest wholly upon the opinions and experiences of MUA practitioners [2] and that the types of spinal conditions most suitable for MUA are without clear-cut consensus [21].

The most recent review paper on MAM for chronic low back pain cites that there is “little evidence” to support the opinion that three MUA procedure doses, administered serially over the same number of days, are necessary to attain the best possible results [2]. This challenges the conventional chiropractic thinking and the more common practice of rendering MUA over three consecutive days. For what may be considered one of the seminal references on the subject of MUA, Krumhansl and Nowacek reported that over a 6 year period a total of 190 MUA procedures were performed on 171 subjects [38]. This would signify that an overwhelming percentage of those patients had received only a single procedure. Elsewhere, some of the chronic low back pain patients within the prospective cohort studies conducted by Kohlbeck, et al. [13] and Palmieri and Smoyak [15] were in receipt of only a single MUA or MAM procedure dose. Perhaps of greatest significance, a consensus document put forth by the American Academy of Osteopathy in 2005 qualifies that the MUA procedure is usually rendered as a single dose [119]. As such, chiropractors should be particularly attentive to individual patient needs rather than summarily presume that three MUA procedure doses would be appropriate or necessary for maximum therapeutic benefit. Also, broader consideration should be given to the potential for a perpetuation of favorable perceptions with treatment approaches that have yet to be substantiated by way of controlled clinical investigation [121].

### Types of MUA treatment

MUJA has been said to be a clinical correlate of MUA [47]. However, it would be an oversimplification to compare MUJA or MUEA with the MUA procedure in general. Differences exist in the type, route and mode of action of the medication agents administered from one procedure to another. Therefore, as for the treatment of any particular clinical diagnosis, the existing base of literature on MUJA/MUEA should not be relied upon as evidence either for or against the efficacy of MUA of the spine via conscious sedation or deep sedation. Moreover, the emerging literature for use of MUA on frozen shoulders and post-operative knees is not generalizable to the spine.

### Components of MUA treatment

Several clinical papers in the earlier MUA literature summarize the results for medium to large case series or offer a generic description about its utility as a successful means of managing patients with pain conditions of the spine [7,17,20,25-28]. For the most part, the principal context of the MUA care outlined in those papers is the provision of mostly a single procedure dose via osteopathic techniques with a hospital stay involving the concomitant administration of one or more types of co-interventions. Consequently, it would be unfitting to conclude that the findings of the studies or commentaries put forth by Clybourne [20], Chrisman, et al. [25], Mensor [26], Morey [7], Rumney [27], Siehl and Bradford [17] and Siehl [28] can be relied upon as evidence of efficacy with contemporary MUA protocols. Significant and numerous variations exist in the overall treatment approach cited in the past versus that of today.

Contemporary MUA protocols lack the support of high quality evidence. It would appear that the experience and observations of a limited number of individuals have shaped the consensus processes by which these protocols have been developed. Suffice it to say there is widespread acceptance of these protocols amongst chiropractors who either perform MUA or refer their patients for the like. Chiropractors have traditionally relied upon published protocols [120] for patient selection purposes as well as for guidance on the parameters for both MUA treatment and the post-MUA phase of care.

Post-MUA rehabilitation is proposed to be an integral and necessary component of MUA care if such treatment is to be of lasting benefit in the restoration of musculoskeletal function [21,35,122]. Following MUA, in order to deter the reformation of vertebral joint and/or myofascial adhesions during the course of healing, both spinal manipulation and a continuance of the stretching/traction type techniques utilized during MUA are to be employed, in part, at each post-MUA follow-up visit to the doctor’s office [5]. With this approach, there would be no legitimate clinical purpose for the provision of MUA if, following its administration, a patient is simply discharged from chiropractic care. Furthermore, the purported benefits of the MUA procedure would theoretically be lost in the instance that a patient returns to office-based care absent the types of manipulation and soft tissue mobilization techniques/maneuvers that could be expected to stress the intersegmental elements to the degree necessary to prevent the reformation of adhesions and to maintain flexibility. Thus, for those who utilize this procedure, the pre-MUA, intra-MUA and post-MUA components of care be must be governed by clinical logical and decision making consistent with the fundamental adhesion-disruption theory upon which MUA has been built.

When chiropractic clinicians do not adhere to a patient-specific chiropractic care regimen leading up to, during, and following MUA of the spine, what develops over time is a patchwork of independent ideas, care methods and technique applications that collectively differ from how the procedure was ever intended to be rendered. This is not to suggest that manipulation of the spine under anesthesia be applied in cookbook fashion for all patients. However, without acknowledgement or consistency of the overall treatment regimen with supportive literature and its theoretical foundation to disrupt and then prevent the reformation of adhesions, the very premise of MUA becomes compromised. By lack of adherence to a more standardized means of selecting and applying all aspects of the procedure, chiropractors may place the future of MUA in jeopardy to the extent that patients who develop a need for the like may no longer have access.

### Professional, ethical and legal considerations for the chiropractic clinician

Beyond the attainment of MUA certification chiropractors should strive to develop a good working knowledge of the substance of the related peer reviewed medical literature. The mere presence of clinical papers in the literature over an 80 year timespan does not summarily connote procedural efficacy. It is the responsibility of the MUA practitioner to understand the nature and scope of the evidence that pertains to the treatment of debilitating musculoskeletal conditions of different body regions. In accordance with the evidence, critical thinking skills and self-governance are necessary to the appropriate utilization and ethical application of the MUA service for each uniquely presenting patient. Nevertheless, it is recognized that lack of protocol/evidence awareness, financial enticement, entrepreneurial motivations and/or clinician assuredness for MUA can contribute to decision making that fails to best meet the needs of individual patients. A case can be made that the potential for indiscriminate use [34] has become a burgeoning issue in need of redress by the chiropractic profession, albeit in all likelihood few advocates of this procedure would be willing to acknowledge this.

Above all, chiropractic must serve the public interest [123]. This requires no explanation. Nevertheless, in its more recent history, it would appear that professionalism in chiropractic has been usurped by commercialism [123]. With broader regard to professional ethics, it has been said that, “*Despite the fact that a chiropractic practice is typically a commercial, for-profit enterprise, the chiropractor is not governed by the dictates of mercantilism but rather by professionalism… Thus, chiropractors, as health professionals, are expected to make recommendations that are in the best interest of the patient, superseding the doctor's pecuniary interests*” [124].

In order that chiropractors may better serve the public, a series of strategic steps were recently proposed for professional renewal in numerous areas including that of ethics [125]. Arguably, this matter has particular relevance to the chiropractic utilization of MUA within the personal injury arena. While purportedly providing an invaluable chiropractic service to those who are experiencing recalcitrant musculoskeletal conditions from an acceleration/deceleration trauma event, there is a seeming emergence of disregard by some in fulfilling basic patient selection criteria for a procedure that is seldom indicated. Accordingly, one might argue that MUA has more recently evolved into a one-size-fits-all treatment approach used in any capacity deemed appropriate by individual clinicians, at times without genuine regard for patient need [68], patient safety [126] or informed consent. This is unacceptable, and should no longer be tolerated by a profession that has yet to overcome negative public perception with regard to honesty/ethics [127] while still lacking cultural authority [123,128,129].

When provider activity surrounding patient selection for MUA lacks clarity, with potential for an ever growing percentage of patients being directed for the like, what might that imply about the efficacy of traditional in-office chiropractic treatment? For more than a century chiropractors have utilized conscious manipulation, adjunctive physiotherapeutic modalities and other conservative care measures to treat spine-based musculoskeletal conditions of varying etiologies. Principally, such treatment is aimed at correcting underlying mechanical dysfunctions or restrictions of spinal/extraspinal articulations and conjoining soft tissues. Care is also rendered for the purpose of accelerating the natural history of healing. Nonetheless, with increased utilization of MUA, particularly when this service is applied in comprehensive fashion after just a few short weeks of office-based care, some chiropractors are exhibiting a behavior that could easily be interpreted by others as an abandonment of routine treatment approaches. Thus, the trend of increasing MUA utilization and/or its metamorphosis into something different from that chronicled throughout the medical literature creates the appearance of a loss of confidence in the efficacy of traditional office-based chiropractic care methods. This is not beneficial for the profession, and could theoretically jeopardize future patient access to the services that are integral to present day office-based chiropractic care.

It is recognized that a lack of evidence of efficacy is not necessarily synonymous with lack of efficacy. Withholding any form of treatment due to the absence of supportive data from randomized controlled trials would be unnecessarily restrictive [130] and likely lead to a state of “therapeutic paralysis” [124]. Moreover, it is acknowledged that scores of testimonials from both doctors and patients have routinely cited the effectiveness of MUA in the treatment of chronic spine pain conditions. Nonetheless, as health care professionals charged with the public trust, chiropractors who perform spinal manipulation under anesthesia, or make referrals for the like, should know and rely upon existing published medical evidence when making clinical decisions for individual patients. Once the influences of anything other than the findings of bona fide clinical investigation or best practice consensus statements enter the patient-care decision making process, particularly with regard to a procedure that has had a history of being controversial [32,35,38,47], the integrity of the doctor patient relationship may become compromised. When educated health care professionals allow their views on patient care approaches to be shaped by testimonials (anecdotal evidence), as if such declarations are somehow akin to research evidence, a doctor’s decision making abilities become compromised and, in essence, are relegated to the level of the laity. This can lead to a breach in the doctor’s fiduciary duties and, in its broadest context, create and then perpetuate an artificial standard of care. This does not serve the public interest.

In contemporary times, pertinent to the rendition of MUA of the spine to individual patients, it is of utmost importance that chiropractors seek to understand the definition of evidence based clinical practice [56]. A patient that has reached clinical endpoint following sufficient trials of in-office manipulation and other modes of conservative care yet is still experiencing significant pain and disability, as measured by way of pain diagrams and disability measurement instruments [5], would be considered a complicated case that may justify consultation for MUA. Considering the deficiencies and differences noted across the existing literature and protocols, it is incumbent upon the MUA provider to substantiate a patient specific clinical rationale concerning the overall breadth of the MUA procedure to be rendered [37]. This pertains to the dysfunctional body region/s qualifying for such treatment and then, perhaps in accordance with the eighty percent threshold improvement criterion [120], the number of procedure doses that follow (whether applied serially [120] or intermittently [119]), if any. Nevertheless, the newly established American Association of Manipulation Under Anesthesia Providers (AAMUAP) alternatively recommends an approach for determining single versus serial MUA on a pre-MUA basis [131].

While the potential for patient complication with MUA exists regardless of the body region under treatment, the relative paucity of reported incidents or published case reports in this area [38,132] appears to indicate that the risk for complication is considerably low with properly selected patients. This matter has been discussed elsewhere [32,34]. Beyond the need for basic medical evidence awareness, chiropractors who regularly utilize MUA in their practices may soon find themselves giving consideration to looming issues of legality and a need to determine treatment alternatives to MUA in managing chronic spine pain patients. This follows a recent Texas court of appeals ruling which classifies MUA in that state as “*a surgical procedure excluded from the statutory scope of chiropractic practice*” [51].

## Conclusion

In the MUA literature there is a long reported history of mostly favorable outcomes. Despite this, the evidence of treatment efficacy remains limited [119], with published studies that are generally weak in their methodological quality [2] and consistently varied across multiple domains which do not permit comparative analysis toward generalization [15]. An earlier chiropractic consensus process resulted in an assigned equivocal rating for MUA (approved for use in clinical practice but requiring further exploration) [133]. Similarly, a more recent evaluation of the clinical utility of MUA in the management of chronic low back pain resulted in no specific recommendations due to a lack of sufficient evidence [2].

In serving the public, chiropractors have a professional obligation to render care in accordance with the best available evidence. In view of the nature and scope of existing research and the outcomes of published professional assessments, the practitioner who is giving consideration to this treatment approach for individual patients should apply caution and tact before proceeding. If MUA is to remain a treatment option for chronic spine pain, it must be reserved for the most stubborn cases and/or under extenuating clinical circumstances. In addition, when appropriate, treatment should be applied to a targeted spinal region as a final resort to attempts at standard conservative treatment measures to alleviate pain and restore function. There is a void of high quality published medical evidence to support the practice of universal MUA treatment of the entire axial spine in the management of a sole regional condition, when there are concomitant but comparatively innocuous complaints/physical findings of vertebral joint pain/dysfunction of other spinal regions.

The three studies which likely represent the current best evidence for MUA via conscious/deep sedation pertain solely to the low back [13,15,23] (Level II evidence). Amongst these studies there are variations in the treating condition reported, the type of intravenous agents used, technique application employed and the number of procedures rendered. These variables pose a clinical challenge for the chiropractor who may be considering this mode of care. In determining the specific components of care to employ, breadth of treatment application and procedure dose, the clinician must rely upon this limited yet diverse evidence in the context of consensus based protocols that have been derived from the experiences and observations of a limited number of individuals. In the near future, chiropractors who perform manipulation under anesthesia may also find themselves confronted with challenges in the scope of practice domain, should the recent judicial battle of Texas [51] widen to other states.

Further research efforts by way of prospective, randomized trials are greatly needed in elevating the quality of research evidence either for or against spinal MUA via conscious/deep sedation and in better defining its role, if any, in the management of explicit spine-based neuromusculoskeletal conditions. Moreover, clinical trials are necessary in qualifying the indications and appropriate parameters of such treatment, including criteria for patient candidacy and optimal procedure dose application. Lastly, comparative studies are needed in clarifying if and under what circumstances MUA may be more efficacious over the long term versus a continuance of traditional office-based chiropractic management procedures or more invasive interventions that lie beyond the scope of chiropractic care. Without these research efforts, the efficacy of MUA relative to other interventions available for chronic spine pain will remain in question.

## Abbreviations

MUA: Manipulation under anesthesia; MAM: Medicine assisted manipulation; MUJA: Manipulation under joint anesthesia; MUEA: Manipulation under epidural anesthesia; MUESI: Manipulation under epidural steroid injection.

## Competing interests

The author declares that he has no conflicts of interest.
